# Loss of ACTL7A causes small head sperm by defective acrosome-acroplaxome-manchette complex

**DOI:** 10.1186/s12958-023-01130-5

**Published:** 2023-09-04

**Authors:** Yini Zhang, Jianan Tang, Xuemei Wang, Yisi Sun, Tianying Yang, Xiaorong Shen, Xinyue Yang, Huijuan Shi, Xiaoxi Sun, Aijie Xin

**Affiliations:** 1 Shanghai Ji Ai Genetics and IVF Institute, Obstetrics and Gynecology Hospital, Fudan University, Shanghai, 200011 China; 2 Shanghai-MOST Key Laboratory of Health and Disease Genomics, NHC Key Lab of Reproduction Regulation,Shanghai Institute for Biomedical and Pharmaceutical Technologies (SIBPT), School of Pharmacy, Fudan University, Shanghai, 200032 China; 3 Shanghai Key Laboratory of Female Reproductive Endocrine Related Diseases, Shanghai, 200011 China

**Keywords:** Actin-like protein 7A, Small head sperm, Acrosome-acroplaxome-manchette complex, Autophagy, Male infertility, Artificial oocyte activation

## Abstract

**Background:**

Actin-like 7 A (ACTL7A) is essential for acrosome formation, fertilization and early embryo development. *ACTL7A* variants cause acrosome detachment responsible for male infertility and early embryonic arrest. In this study, we aim to explore the additional functions of ACTL7A beyond the process of acrosome biogenesis and investigate the possible underlying mechanisms.

**Methods:**

Nuclear morphology analysis was used to observe the sperm head shape of *ACTL7A*-mutated patients. *Actl7a* knock-out (KO) mouse model was generated. Immunofluorescence and transmission electron microscopy (TEM) were performed to analyze the structure of spermatids during spermiogenesis. Tandem mass tags labeling quantitative proteomics strategy was employed to explore the underlying molecular mechanisms. The expression levels of key proteins in the pathway were analyzed by western blotting. Intracytoplasmic sperm injection (ICSI)-artificial oocyte activation (AOA) technology was utilized to overcome fertilization failure in male mice with a complete knockout of *Actl7a*.

**Results:**

The new phenotype of small head sperm associated with loss of ACTL7A in patients was discovered, and further confirmed in *Actl7a*-KO mice. Immunofluorescence and TEM analyses revealed that the deletion of ACTL7A damaged the formation of acrosome-acroplaxome-manchette complex, leading to abnormalities in the shaping of sperm heads. Moreover, a proteomic analysis of testes from WT and *Actl7a*-KO mice revealed that differentially expressed genes were notably enriched in PI3K/AKT/mTOR signaling pathway which is strongly associated with autophagy. Inhibition of autophagy via PI3K/AKT/mTOR signaling pathway activation leading to PDLIM1 accumulation might elucidate the hindered development of manchette in *Actl7a*-KO mice. Remarkably, AOA successfully overcame fertilization failure and allowed for the successful production of healthy offspring from the *Actl7a* complete knockout male mice.

**Conclusions:**

Loss of ACTL7A causes small head sperm as a result of defective acrosome-acroplaxome-manchette complex via autophagy inhibition. ICSI-AOA is an effective technique to rescue male infertility resulting from ACTL7A deletion. These findings provide essential evidence for the diagnosis and treatment of patients suffering from infertility.

**Supplementary Information:**

The online version contains supplementary material available at 10.1186/s12958-023-01130-5.

## Background

Spermatogenesis is a critical process responsible for generating mature male gametes in mammals. It can be categorized into three major phases: spermatogonia mitosis, spermatocytes meiotic division, and the final differentiation phase known as spermiogenesis [1]. During spermiogenesis, the round spermatids undergo substantial changes, including condensation of nuclear chromatin, formation of acrosome, elongation of the sperm head and assembly of the flagella [1, 2]. The acrosome-acroplaxome-manchette complex plays a critical role in the shaping of spermatid head [3]. A large number of specific proteins make up this complex, and any defects may result in the malformation of sperm, eventually infertility. Knockout of several genes involved in acrosome biogenesis leads to globozoospermia [4–6]. Acroplaxome, a cytoskeletal scaffold containing F-actin and Keratin 5, anchors the developing acrosome to the nuclear envelope [7]. Defects in acroplaxome structure result in acrosome detachment [8]. Manchette is a temporary skirt-like structure that surrounds the elongating spermatid head exclusively during the elongation process of spermiogenesis. It acts as a platform providing microtubular tracks for the efficient transport of vesicles and proteins essential to sperm development. Severe impairment in the formation of manchette causes amorphous head shape and flagellar defects [9].

Actin like protein 7 A (ACTL7A) belongs to a highly conserved actin-related proteins (ARPs) family which has 17–45% sequence identity with actin. Our team first reported the disruption in ACTL7A (c.733G > A, p.Ala245Thr) causes acrosome defects responsible for early embryonic development [10]. Subsequently, several reports had shown that different variants in *ACTL7A* were associated with male infertility, displaying different degrees of acrosome abnormalities [11–14]. The expression level of ACTL7A on sperm is strongly related to fertilization outcomes of assisted reproductive technologies (ART) [14]. Latest reports have demonstrated that ACTL7A interacts with various cytoskeletal proteins and is an indispensable protein for subacrosomal-associated F-actin formation, acrosomal anchoring and male fertility [15].

In the present study, we discovered a new phenotype of small head sperm associated with loss of ACTL7A in patients. *Actl7a* knock-out (KO) mouse model was constructed to further explore the function and mechanism of ACTL7A.The results demonstrated that in addition to causing acrosome detachment, deletion of ACTL7A led to small head sperm due to the damaged acrosome-acroplaxome-manchette complex. In a further attempt to address the molecular mechanism, we performed the mass spectrometry on testes and found that inhibition of autophagy via PI3K/AKT/mTOR activation resulted in the accumulation of PDZ and LIM domain 1 (PDLIM1), leading to the defective manchette. Moreover, intracytoplasmic sperm injection (ICSI)-artificial oocyte activation (AOA) technology could overcome fertilization failure, allowing *Actl7a*-KO mice to produce offspring. This indicates that ICIS-AOA is still effective in sperm with no ACTL7A protein expression, which provides compelling evidence for clinicians to choose the optimal treatment for patients.

## Materials and methods

### Ethical statement

All protocols for human studies were approved by the Ethics Committee of the Shanghai Ji Ai Genetics and IVF Institute (JIAI E 2018-21). Written informed consents were obtained from subjects participating in this study. All animal experiments were approved by the Animal Care Committee of the Shanghai Institute for Biomedical and Pharmaceutical Technologies and conducted in accordance with standard ethical guidelines of the International Review Board and/or Institutional Animal Care and Use Committee guidelines.

### Generation of *Actl7a* knock-out (KO) mice

*Actl7a*-KO (C57BL/6) mice were generated by CRISPR-Cas9 technology as previously described [10]. The single guide RNAs (sgRNAs) (5’-ACGGGGAGGCTAGACTACGC-3′), Cas9 mRNA and single-stranded oligonucleotides (ssODNs) (5’-TATGAGGGTTATCCTTTGCCCAGCATCACGGGGAGGCTAGACTACACAGGTTCTGACCTAACGACCTACCTGATGAACCTGATGAACAA-3′) were injected into the cytoplasm of zygotes. The zygotes survived without any abnormalities after being put into the CO_2_ incubator. Subsequently, embryos were implanted into pseudo-pregnant C57BL/6 females to generate offspring. The genotyping primers used were as follows: 5′-GAGAAGTACGCCGAGATGCT-3′ (forward) and 5′-CTCCTGACCCAGACGGATCT-3′ (reverse).

### Assessment of fertility

A male was mated with two females to determine the fertility of the *Actl7a*-KO male mice. Females were checked for the presence of vaginal plugs, indicating pregnancy, and then separated from males. The number of mice achieving a pregnancy and the litter sizes were recorded.

### Assessment of sperm motility

Sperm samples were collected from mouse cauda epididymis into human tubal fluid (HTF) medium drop and incubated at 37 °C. A computer-assisted sperm analysis program HTM-TOXIVOS (Hamilton-Thorn Research, MA) was used for sperm functionality analysis.

### Histological analysis

The testes and epididymis tissues were dissected and fixed in paraformaldehyde (PFA) at 4 °C overnight. Fixed tissues were dehydrated by gradually soaking in alcohol and xylene, and then embedded in paraffin. Before staining, Sect. (4-µm-thick) underwent dewaxing, benzene removal, and hydration. Images were acquired using an Olympus BX60 microscope.

### Nuclear morphology analysis

To analyze sperm head morphology, human sperms collected by masturbation and mice sperms isolated from the cauda epididymis were fixed with 4% PFA, followed by washing three times with PBS. Sperms were collected by centrifugation at 500 × g for 5 min and resuspended in PBS. Sperm cell suspension was spread onto a glass slide, and then air-dried. Finally, slides were counterstained with DAPI containing mounting media. Confocal microscopy images were captured with Nikon A1 + Confocal Microscope System (Tokyo, Japan). ImageJ plug-in “Nuclear Morphology Analysis version 1.15.1” was used for morphology analysis.

### Immunofluorescence

Adult mouse testes were removed from the tunica albuginea with sterile forceps, and seminiferous tubules were minced with scissors to release cells in PBS and incubated at 37 °C for 30 min. The final suspension was filtered through a mesh cellular filter with a pore size of 40 μm to obtain a single-cell suspension. Sperms were collected by centrifugation and fixed with 4% PFA at room temperature for 15 min incubation. After washing three times with PBS by centrifugation, the suspension of fixed testicular cells was mounted onto polylysine-coated slides and allowed to air dry completely. For immunofluorescence assay, the smear was permeabilized with 0.2% Triton X-100 at 37 °C for 15 min, blocked for 1 h with 10% bovine serum albumin (BSA), and then incubated with mouse anti-α-tubulin (1:1000) (Cat# T-6199, Sigma) in blocking solution at 4 °C overnight. After washing, fluorescence-conjugated secondary antibody, goat anti-mouse Alexa Fluor 647 (1:1000) (Cat# A-21,235, Invitrogen), was applied at room temperature for 1 h. The slides were further incubated with Alexa Fluor 555-Phalloidin (1:100) (Cat# SB-YP0060, Share-bio) for detecting sperm acroplaxome and Alexa Fluor 488–conjugated PNA (20 g/ml; Molecular Probes, CA, USA) for detecting sperm acrosome in dark. Finally, nuclei were counterstained with 1 µg/ml Hoechst 33,258. Confocal images were taken as described above.

### Transmission electron microscopy (TEM)

WT and *Actl7a*-KO adult mice were sacrificed by cervical dislocation. The testicular tissues were sliced into small pieces of 1 mm^3^ in size after being isolated quickly and fixed in 2.5% glutaraldehyde at 4 °C overnight. Tissues were rinsed three times with 0.1 M phosphate buffer for 15 min intervals and post-fixed in 1% osmium tetroxide at room temperature for 1.5 h. After three additional washes with double distilled water for 10 min intervals, the samples were dehydrated through an ethanol series including 30, 50, 70, 80, and 95% ethanol for 10 min each procedure, followed by 100% acetone twice at 15 min intervals. The samples were infiltrated with a 1:1 mix of acetone: Epon 812 for 2 h, and then embedded in pure Epon 812 at 37 °C overnight and at 60 °C for 48 h to polymerize. Ultrathin sections were cut, mounted on copper grids and stained with uranyl acetate followed by lead citrate. TEM (Tecnai-10, Philips) was used for observation and image capturing.

### Mass spectrometry analysis

Testicular proteins of three mice from each of the WT and *Actl7a*-KO groups were extracted and digested for tandem mass tags (TMT) labeling and liquid chromatography-tandem mass spectrometry (LC-MS/MS) analysis. TMT-labeled peptides were resuspended in 0.1% formic acid. Mobile phase B was 80% acetonitrile containing 0.1% formic acid. Peptides were eluted with a gradient of mobile phase B which increased from 2 to 20% for 170 min, from 20 to 30% for 4 min, from 30 to 100% for 1 min, and to 100% for 5 min. The flow rate was 300 nL/min. Peptide fractions were analyzed by an Orbitrap Exploris ™ 480 mass spectrometer (Thermo Fisher Scientific) coupled with an Easy-nLC1200 system (Thermo Fisher Scientific). MS raw data were processed using Proteome Discoverer 2.4 (Thermo Fisher Scientific).

### Protein extraction and western blotting analysis

Total protein was extracted from testicular tissues of WT and *Actl7a*-KO mice which were lysed and homogenized with cold RIPA containing fresh phenylmethylsulfonyl fluoride and protease inhibitor cocktail. Testicular extracts were heated at 95 °C for 10 min in sample buffer. Protein concentrations in the supernatants were measured using the BCA protein assay (Pierce™ BCA Protein Assay Kit). Then, equal amounts of protein samples were separated on a 10% Tris-glycine gel and transferred onto a polyvinylidene fluoride (PVDF) membrane by the semidry method. After that, the PVDF membrane was blocked with 5% BSA in Tris-buffered saline with Tween 20 (TBST) at room temperature for 1 h, and then incubated with relevant proper primary antibodies at 4 °C overnight. Next, the membrane was washed three times with TBST for 10 min, incubated with secondary antibody conjugated to horseradish peroxidase at room temperature for 1 h, and washed three times with TBST for 10 min again. Ultimately, protein bands were visualized with an enhanced chemiluminescence (ECL) kit. All expressions of proteins were compared with control β-actin, and quantitative analysis was performed by ImageJ software. The antibodies used in western blotting were as follows: p-AKT (1:2000) (Cat# 4060, Cell Signaling), AKT (1:1000) (Cat# 4691, Cell Signaling), p-PI3K (1:1000) (Cat# 4228, Cell Signaling), PI3K (1:1000) (Cat# 4257, Cell Signaling), p-mTOR (1:1000) (Cat# AP0094, ABclonal), mTOR (1:1000) (Cat# a2445, ABclonal), β-actin (1:2500) (Cat# ab133626, Abcam), SQSTM1 (1:1000) (Cat# A19700, ABclonal), LC3B (1:1000) (Cat# A19665, ABclonal).

### Immunohistochemistry

Paraffin-embedded tissue sections were dewaxed in xylene and rehydrated using a graded series of alcohol. To eliminate internal peroxidase activity, 3% hydrogen peroxide was added to the sections. Next, sections were boiled for 15 min in sodium citrate buffer for antigen retrieval. After blocking with goat serum for 30 min, the sections were incubated with primary antibodies at 4°C overnight, followed by staining with the HRP-conjugated secondary antibody. After washing, the sections were stained with 3,3’-diaminobenzidine (DAB), counterstained with hematoxylin and observed under light microscopy.

### ICSI in mice

Five to six weeks-old female B16D2F1 (C57BL/6 × DBA2) hybrid mice were superovulated by intraperitoneal injecting 7.5 international units (IU) of pregnant mare serum gonadotropin (PMSG) and following 7.5 IU of human chorionic gonadotropin (hCG) 48 h later. Fourteen hours later, oocyte-cumulus complexes (OCC) were obtained by dissecting the ampulla of the oviduct. The sperm samples of WT and *Actl7a*-KO mice were collected from caudal epididymis, transferred into the HTF medium and incubated at 37 °C to allow mature sperms to swim out.

For ICSI, MII-arrested eggs were collected from the oviduct of females by culturing OCC at room temperature for 5 min in KSOM containing hyaluranidase to digest and remove the cumulus cells. Next, sperm heads were injected into oocytes using a piezo-driven micromanipulator. All oocytes were further cultured in KSOM medium at 37 °C with 5% CO_2_. The two-cell zygotes were then surgically transplanted into the oviduct of pseudo-pregnant ICR female mice. Finally, viable pups were obtained via natural labor.

### Artificial oocyte activation

AOA was performed as previously described [10]. After ICSI, oocytes were activated in KOSM medium containing 5 mM SrCl_2_ (Sigma-Aldrich, St Louis, MO, USA) for 6 h. Afterward, oocytes were rinsed thoroughly, transferred to fresh KSOM medium and cultured at 37 °C with 5% CO_2_.

### Statistical analysis

Statistical analysis was performed using GraphPad Prism 8 software with one-way analysis of variance (ANOVA) or two-tailed Student’s *t* test. All data were generally presented as mean ± standard error of the mean (SEM). The results were considered statistically significant for *P*-value < 0.05.

## Results

### Loss of ACTL7A causes small head sperm

**Fig. 1:**
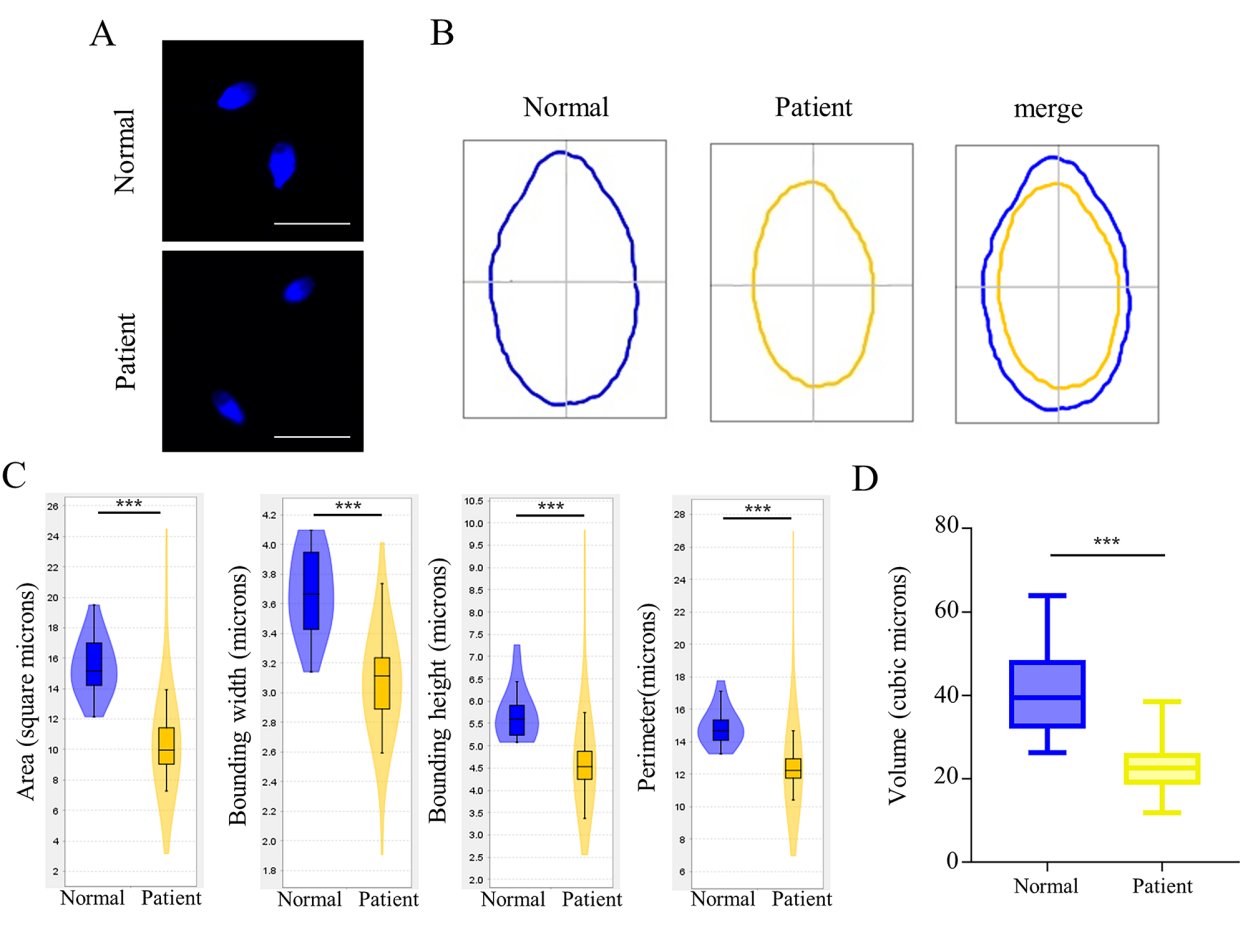
Sperm head morphology analysis of sperm cells in the normal individual and the *ACTL7A*-mutated patient. (**A**) Sperm nuclei stained with DAPI in the normal individual and the patient. 82% of sperm in *ACTL7A*-mutated patient showed smaller head compared with normal sperm. (**B**) Head morphology of sperm cells in the normal individual and the patient. (**C**) Area, bounding width, bounding height and perimeter of sperm head in the normal individual and the patient. (**D**) The elliptical volume of sperm head in the normal individual and the patient. Scale bars: 10 μm. ***P < 0.001

Our previous study had reported infertile patients with a homozygous missense mutation of *ACTL7A* (c.733G > A, p.Ala245Thr), leading to extremely reduced expression of ACTL7A protein [10]. Using geometric morphometric analysis, we observed 82% of sperm in the *ACTL7A*-mutated patient showing smaller head size compared with normal sperm (Fig. 1A and B). The area, perimeter, bounding width and height of sperm head were all affected (Fig. 1 C). The human sperm head was approximately oval-shaped. We further conducted a statistical analysis on the elliptical volume of sperm. Compared with the normal individual, the *ACTL7A*-mutated patient had significantly smaller sperm head size (Fig. 1D).

**Fig. 2:**
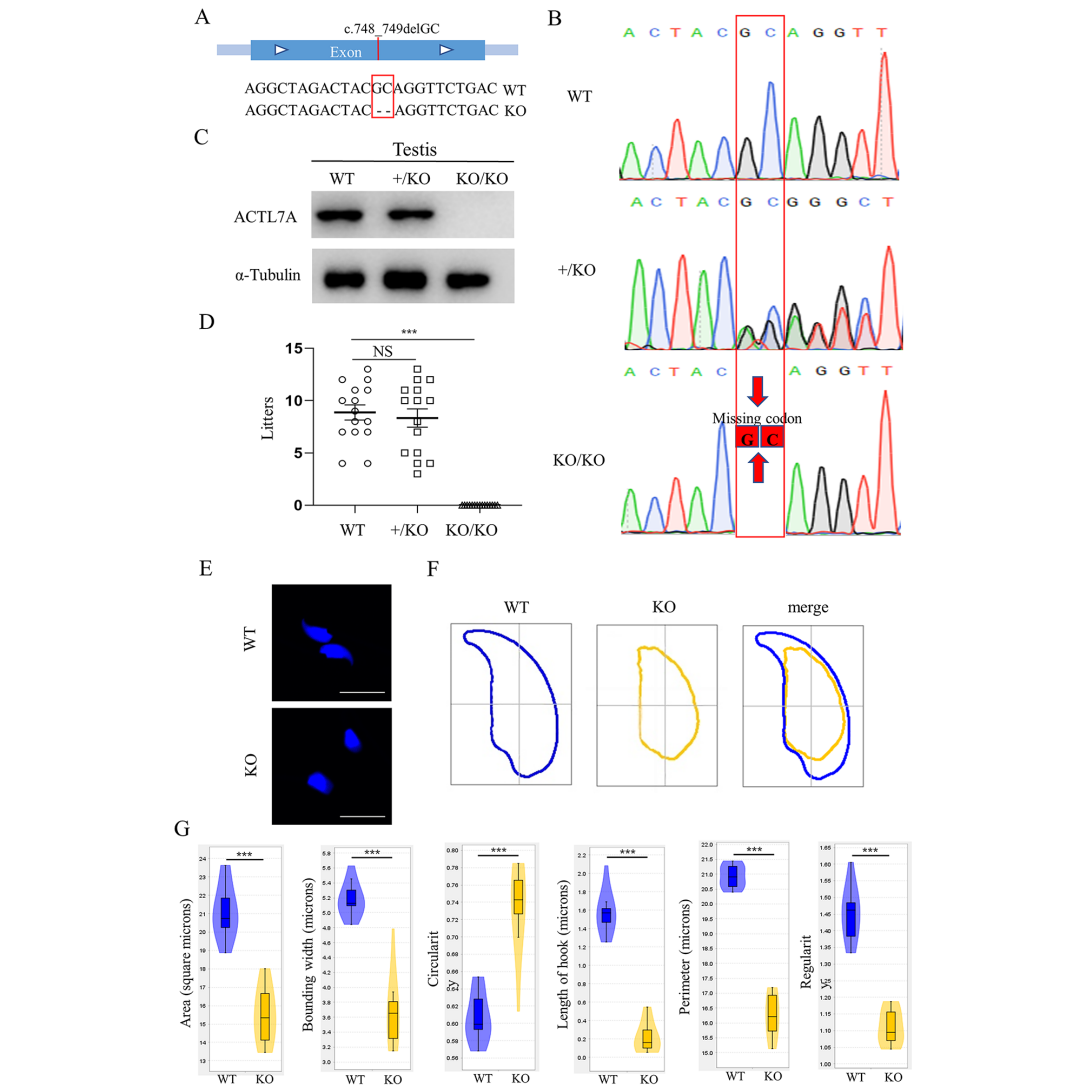
Homozygous knock-out mutation in *Actl7a* leads to male infertility and abnormal sperm head morphology in the mouse model. (**A**) Schematic illustration of strategies for the generation of *Actl7a* knock-out (KO) mice using CRISPR/Cas9 technology. 2 bp of *Actl7a* were deleted from Exon. (**B**) Genotyping of the constructed *Actl7a* knockout mutation in mice. WT, wild type; +/KO, heterozygous mutation; KO/KO, homozygous mutation. (**C**) The ACTL7A protein was completely absent in the testes of *Actl7a*-KO mice. α-tubulin was used as a loading control. (**D**) The *Actl7a*-KO mice were completely infertile. The fertility assessment experiments were performed among the male WT (WT; n = 5) mice, the male heterozygous (+/KO; n = 5) mutation mice and the male homozygous (KO/KO; n = 5) mutation mice. One-way ANOVA, ***P < 0.001; NS, not significant. Data are means ± SEM. (**E**-**G**) Sperm head morphology analysis of sperm cells in WT and *Actl7a*-KO mice. (**E**) Sperm nuclei stained with DAPI in WT and *Actl7a*-KO mice. 75% of the mutant sperm lacked the hook-like structure and showed smaller head compared with normal sperm. (**F**) Head morphology of sperm cells in WT and *Actl7a*-KO mice. (**G**) Area, bounding width, circularity, length of hook, perimeter, and regularity of sperm head in WT and *Actl7a*-KO mice

To explore the potential new function of *ACTL7A* gene, we constructed an *Actl7a*-KO mouse model using CRISPR/Cas9 gene editing technology combined with microinjection technology. The guanine and cytosine, respectively, at the 748th and 749th position of the *Actl7a*-coding region, were deleted, leading to a frame-shift mutation (Fig. 2A). The representative Sanger sequence images for genotyping of *Actl7a* knockout mice are displayed in Fig. 2B. The deletion of two bases caused the complete absence of ACTL7A protein in the testes of *Actl7a*-KO mice (Fig. 2 C). In addition, *Actl7a*-KO mice exhibited completely male sterile (Fig. 2D). Also, there were no statistically significant differences observed in the number of sperms, sperm motility and sperm progressive motility between *Actl7a*-KO mice and WT mice (FIGURE S1A). Histological examination by H&E staining displayed the normal architecture of seminiferous tubules and epididymis in *Actl7a*-KO mice (FIGURE S1B).

We further analyzed the head morphology of mature sperm in *Actl7a*-KO mice. Specifically, 75% of mutant sperm exhibited an absence of the hook-like structure. Consistent with the observations in the human *ACTL7A*-mutated sample, the head of sperm in *Actl7a*-KO mice also revealed reduced size (Fig. 2E and F). Detailed analysis showed that the sperm of *Actl7a*-KO mice had significant alterations in area, bounding width, circularity, length of hook, perimeter and regularity (Fig. 2G).

### Disrupt acrosome-acroplaxome-manchette complex causes small head sperm in *Actl7a*-KO mice

**Fig. 3:**
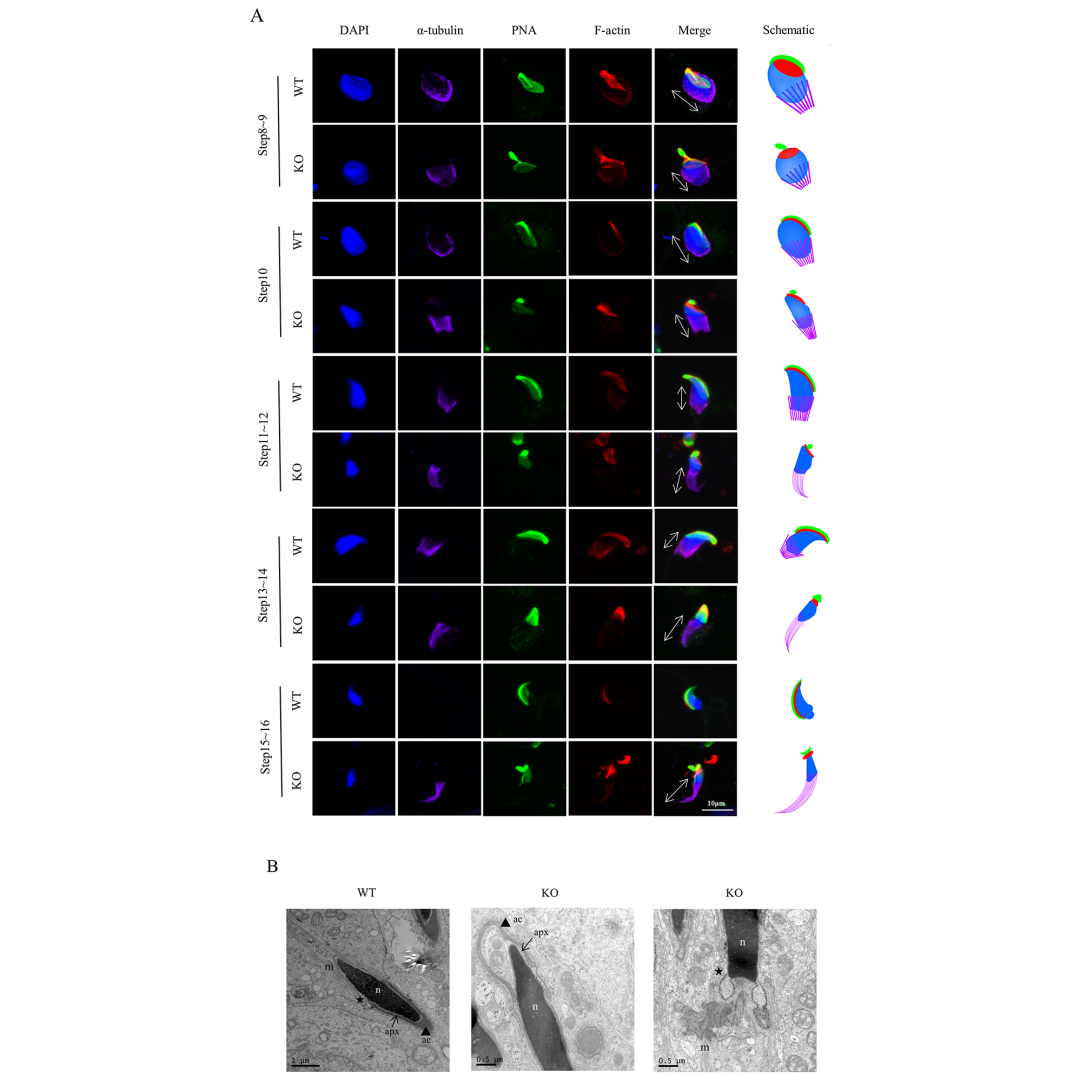
Damaged acrosome-acroplaxome-manchette complex causes small head sperm in *Actl7a*-KO mice. (**A**) Testicular germ cells prepared from WT and *Actl7a*-KO adult mice were stained using the antibody against α-tubulin (purple), PNA (green) and phalloidin (red) to show the manchette, acrosome and acroplaxome formation, respectively. The nucleus was stained with DAPI (blue). Different steps of spermiogenesis were depicted on the basis of co-immunofluorescence staining. The distance from the perinuclear ring of the manchette to the caudal side of the head is indicated by double-headed arrows. Scale bars: 10 μm. (**B**) TEM analysis in WT and *Actl7a*-KO mice shows spermatids at steps 10 ~ 11. *Actl7a*-KO spermatids show acrosome detachment and loosened acroplaxome. The manchette seems abnormally elongated and malformed in *Actl7a*-KO mice. N, nucleus; ac (arrowhead), acrosome; apx (arrow), acroplaxome; m, manchette; asterisk, perinuclear ring. Scale bars: 1 μm and 0.5 μm

Acrosome-acroplaxome-manchette complex plays an essential role in the shaping of spermatid head. To investigate the acrosome-acroplaxome-manchette complex structure of *Actl7a*-KO mice, multiplex immunofluorescence staining was performed (Fig. 3A). PNA, phalloidin and anti-α-tubulin antibody were used to identify acrosome, acroplaxome and manchette, respectively. The nuclei of spermatids were stained with DAPI. In WT spermatids, the acroplaxome present in the subacrosomal space linked the acrosome to the nuclear membrane [7]. During spermiogenesis, the manchette, a transient skirt-like structure, moved toward the tail region and disassembled once nuclear elongation and condensation were complete [16]. However, the acrosome-acroplaxome-manchette complex appeared to have various forms of changes in *Actl7a*-KO mice. Deletion of ACTL7A caused a loosened acroplaxome structure with disorganized distribution and acrosome detachment. The manchette of *Actl7a*-KO spermatid generally appeared normal from steps 8 to 10, however, it gradually elongated from steps 11 to 16 in an aberrant manner and did not disappear after shaping the sperm head at step 16 during spermiogenesis (Fig. 3A).

To further analyze the structure of acrosome-acroplaxome-manchette complex in detail, the TEM experiment was performed (Fig. 3B). In *Actl7a*-KO mice, the acrosome and the acroplaxome presented ectopic positions. The manchette became abnormally longer in elongating spermatids, and its prenuclear ring aberrantly localized the distal end of spermatid head.

These results indicated that the deletion of ACTL7A damaged the formation of acrosome-acroplaxome-manchette complex during spermiogenesis responsible for abnormalities of the sperm head.

### Inhibition of autophagy via PI3K/AKT/mTOR signaling pathway activation leads to defective manchette development in *Actl7a*-KO mice

Loss of ACTL7A causes defective acrosome-acroplaxome-manchette complex. Owing to ACTL7A being a cytoskeletal protein belonging to the acroplaxome, the deficiency of ACTL7A inevitably causes structural disruption of acroplaxome and acrosome. Nevertheless, the impact of ACTL7A on the manchette structure remains unknown due to its different position.

**Fig. 4:**
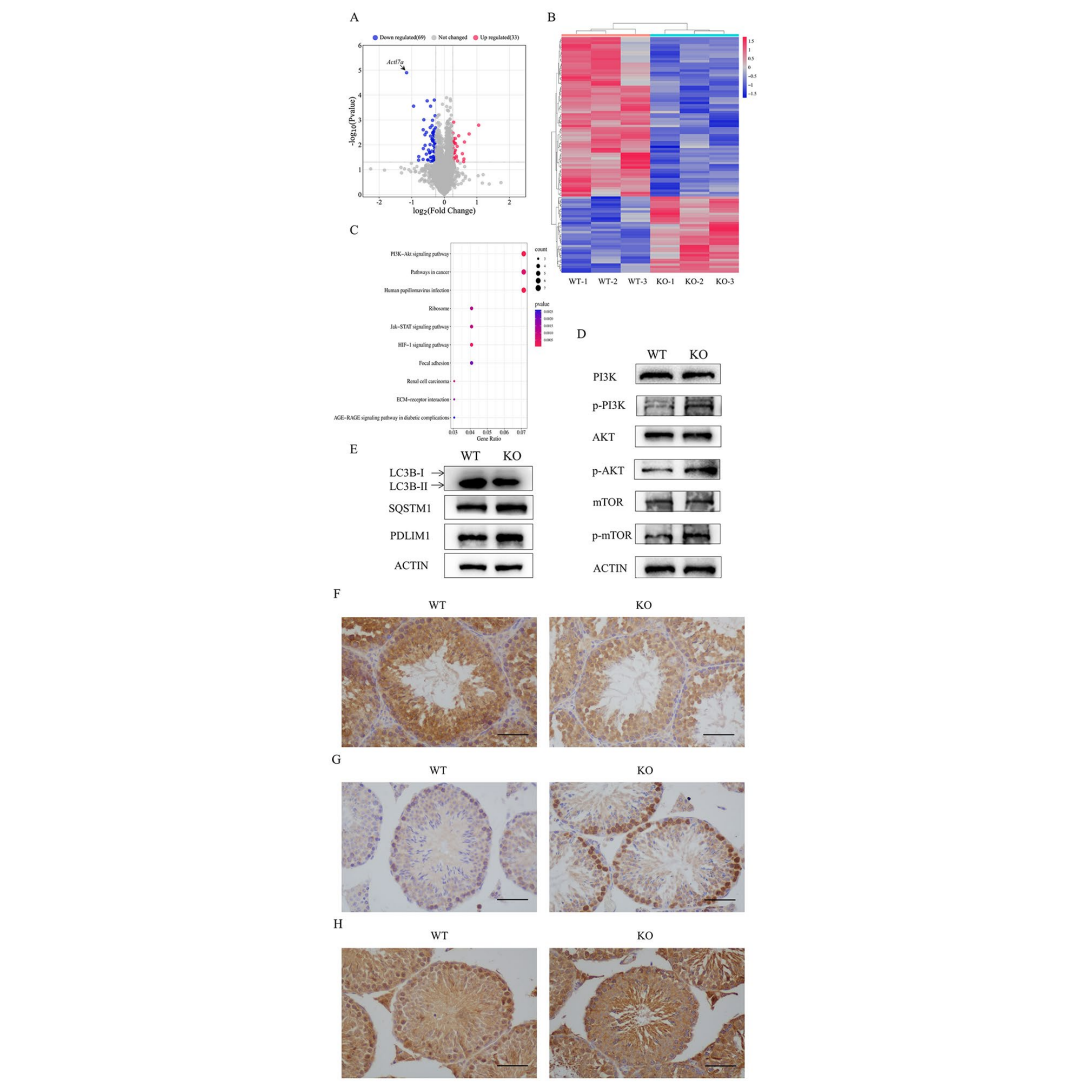
Inhibition of autophagy via PI3K/AKT/mTOR signaling pathway activation causes manchette defects in *Actl7a*-KO mice. (**A**-**C**) The proteome data identified differentially expressed genes (DEGs) in testes from WT and *Actl7a*-KO male mice. (**A**) DEGs are represented as a volcano plot. Red dots represent significantly up-regulated genes (n = 33), and blue dots represent significantly down-regulated DEGs (n = 69) compared with WT control. X-axis: log2 fold change (log2 FC), Y-axis: –log10 (*P*-value). (**B**) Heatmap analysis of DEGs shows the hierarchical clustering between two groups. Each row represents a single gene. Red represents high gene expression, and blue represents low gene expression. (**C**) KEGG enrichment analysis displays the top ten pathways of DEGs. X-axis: gene radio, Y-axis: KEGG pathways. The dot size is proportional to gene count, and *P*-value is indicated by color. (**D**) WB using PI3K, p-PI3K, AKT, p-AKT, mTOR, and p-mTOR antibodies on protein extracts from WT and *Actl7a*-KO testes. (**E**) WB using LC3B-I/II, SQSTM1/p62 and PDLIM1 antibodies on protein extracts from WT and *Actl7a*-KO testes. β-actin was used as a loading control. IHC using (**F**) LC3B-I/II, (**G**) SQSTM1/p62, and (**H**) PDLIM1 antibodies on WT, and *Actl7a*-KO testes sections. Scale bars: 50 μm

To explore the mechanism of ACTL7A deficiency leading to disruption of acrosome-acroplaxome-manchette complex, testis proteins of WT and *Actl7a*-KO male mice were analyzed by TMT labeling quantitative proteomics strategy. 102 differentially expressed genes (DEGs) were identified. Among these, 69 genes were down-regulated and 33 genes were up-regulated in *Actl7a*-KO mice (cutoffs of *P*-value < 0.05 and FC ≥ 1.2 or ≤ -1.2; Fig. 4A). The expression profiling was illustrated in a heatmap with hierarchical clustering (Fig. 4B). KEGG enrichment analysis was conducted on the DEGs, revealing significant enrichment in ten pathways (Fig. 4 C). Of those, the PI3K-AKT signaling pathway enriched the most numerous genes and showed the strongest statistical significance (Fig. 4 C). PI3K/AKT/mTOR signaling pathway was a critical negative regulator controlling autophagic activity in mammalian [17]. It was also previously reported that autophagy facilitated cytoskeleton organization by degrading PDLIM1, a negative regulator of cytoskeleton organization, and accumulation of PDLIM1 led to the disorganization of the cytoskeleton, including manchette [18]. The above evidence suggested an interesting connection among *Actl7a* knockout, autophagy and manchette.

To verify the potential molecular mechanism of abnormal manchette in *Actl7a*-KO mice, the expression levels of key proteins in the PI3K/AKT/mTOR pathway were analyzed by western blotting. The results showed that phospho-AKT, phospho-P13K and phospho-mTOR protein levels increased obviously in testes of *Actl7a*-KO mice, while the total protein levels (AKT, PI3K and mTOR) remained unchanged (Fig. 4D). This indicated the activation of PI3K/AKT/mTOR signaling pathway. LC3B-I/II is a core protein in autophagy formation which can estimate the level of autophagy. SQSTM1/ p62, a selective autophagy receptor and degraded by autophagy, is used to assess the autophagic protein degradation rate [19, 20]. Western blotting and immunohistochemistry (IHC) analysis showed that the LC3B-I/II decreased, while SQSTM1 protein accumulated in *Actl7a*-KO mice (Fig. 4E, F and G). This finding elucidated that the activation of the PI3K/AKT/mTOR signaling pathway resulted in the inhibition of autophagy. Also, we detected the protein level of PDLIM1 by western blotting and IHC (Fig. 4E and H). And results showed that PDLIM1 was obviously accumulated in *Actl7a*-KO mice. Taken together, these data demonstrated that the loss of ACTL7A resulted in autophagy inhibition via PI3K/AKT/mTOR signaling pathway activation and further accumulation of PDLIM1, which was responsible for disrupting cytoskeleton assembly, including defective manchette of spermatids in *Actl7a*-KO mice.

### *Actl7a*-KO mice can have offspring by ICSI-AOA

**Fig. 5:**
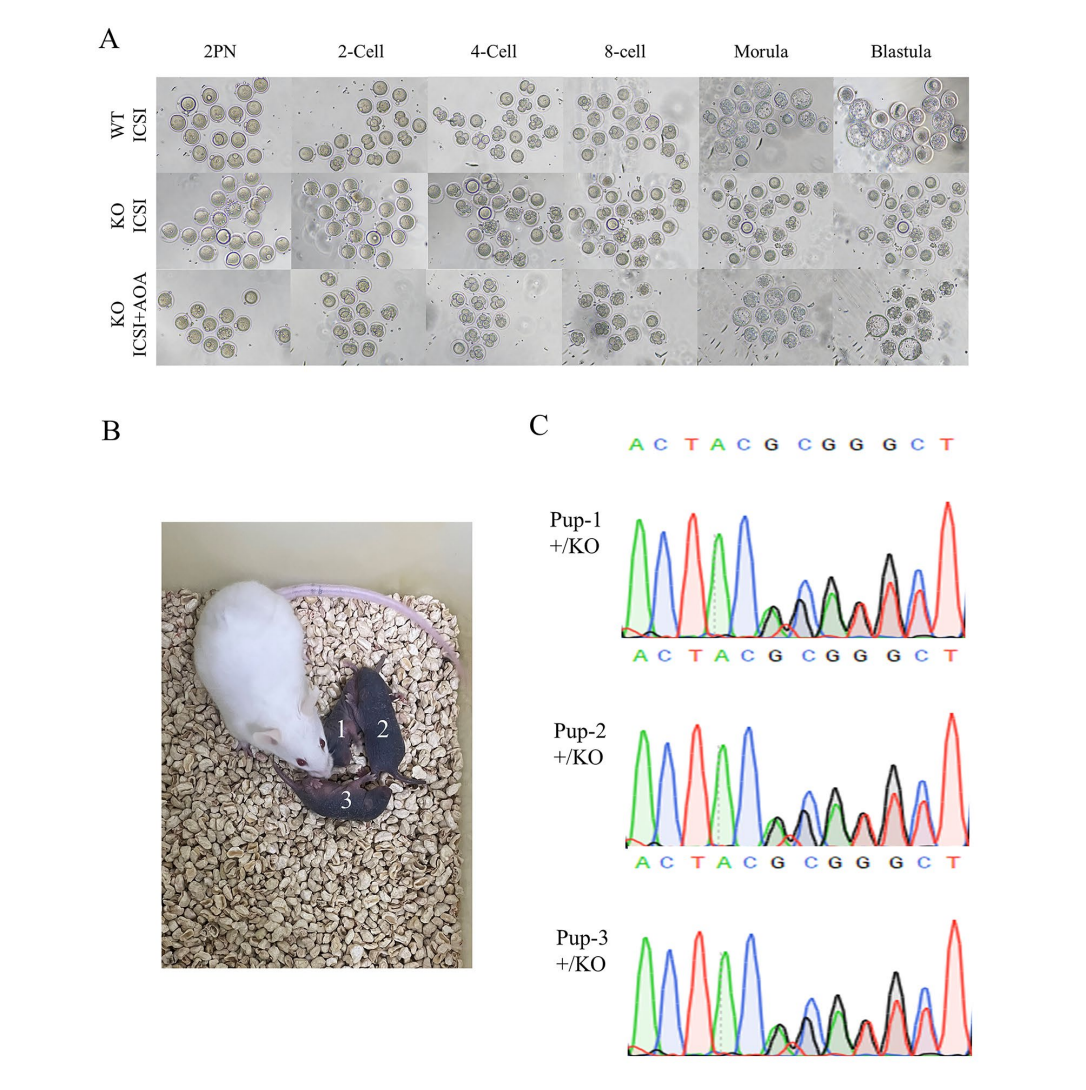
The infertility of *Actl7a*-KO mice can be successfully rescued by ICSI-AOA. (**A**) The oocytes were fertilized by the sperms from the *Actl7a*-KO mice after AOA. The sperms of the *Actl7a*-KO mice failed to fertilize the normal oocytes by ICSI (lane 2). However, the combination of ICSI and AOA enabled the sperms of the *Actl7a*-KO mice to successfully fertilize the oocytes and develop into blastocysts (lane 3). (**B**) The offspring of the *Actl7a*-KO mice were born after ICSI-AOA. (**C**) Genotyping for the newborn pups of *Actl7a*-KO mice. +/KO, heterozygous mutation

It has been reported that ICSI-AOA can successfully rescue infertility caused by mutated *Actl7a*/*ACTL7A*, including missense mutation [10] and compound heterozygous variants [12]. However, it is unknown whether ICSI-AOA is effective in *Actl7a*-KO mice with the complete absence of ACTL7A protein. So, we attempted this technique with strontium chloride (SrCl_2_). ICSI-AOA could make sperms from *Actl7a*-KO mice successfully fertilize with oocytes and develop into blastocysts (Fig. 5A). To further investigate whether AOA could enable the *Actl7a*-KO mice to have offspring and exclude the possibility of parthenogenetic activation, we transplanted two-cell embryos into pseudo-pregnant mice and successfully obtained newborn pups (Fig. 5B). The genotyping results indicated that all pups were heterozygotes (Fig. 5 C). This indicated that ICSI-AOA remains an effective technique to rescue infertility caused by defective ACTL7A, even in the complete absence. These findings provide essential evidence for the diagnosis and treatment of patients suffering from infertility.

## Discussion

In this study, a new phenotype characterized by small head sperm was observed in the patient with a homozygous *ACTL7A* mutation (c.733G > A, p.Ala245Thr), which was the first reported case of an *ACTL7A* variant by our research team [10]. We constructed an *Actl7a*-KO mouse model and discovered a similar phenotype due to the deletion of ACTL7A. The analysis of subcellular structures in spermatids demonstrated that the damaged acrosome-acroplaxome-manchette complex was the main reason for small head sperm in *Actl7a*-KO mice. Further investigation into the molecular mechanism indicated that loss of ACTL7A resulted in inhibition of autophagy via the PI3K/AKT/mTOR signaling pathway, which was responsible for the failure of manchette development in *Actl7a*-KO mice. Additionally, ICSI-AOA remained an effective technique to rescue infertility caused by the complete deletion of ACTL7A.

*ACTL7A* with different mutations leads to male infertility [10–12]. The genetic analysis of *ACTL7A* can be an efficient method to identify previously neglected cases of unexplained male infertility. However, genetic testing is both costly and time-consuming, and requires an additional blood sample. Here, we found a new phenotype of small head sperm due to ACTL7A deletion, which had a statistically significant difference. Measuring sperm volume can be another effective method for determining infertility owing to defective ACTL7A. The method is fast, inexpensive, technically simple and not requiring a blood draw, rendering it convenient and helpful for clinical diagnosis and evaluation.

During spermiogenesis, one of the most noticeable changes is that the round spermatids develop into elongated mature sperms. It has been reported that mice with sperms exhibiting diverse unusual head shapes are infertile, suggesting that the typical head shape of sperm is fundamentally vital for the process of sperm fertilization [21, 22]. For acrosome biogenesis, Golgi-derived proacrosomal vesicles gradually accumulate, coalesce and fuse together to form an acrosomal vesicle bound to the nuclear envelope which attaches to the acroplaxome before finally developing into the acrosome [23]. Meanwhile, the acroplaxome serves as a link between the developing acrosome and the spermatid nucleus. The manchette, a microtubule- and actin-containing structure, begins to assemble with the initiation of the spermatid nucleus elongation [16, 24]. It is important that the marginal ring of the acroplaxome and the perinuclear ring of the manchette contract to deform the acrosome and nucleus. Here, we found that the acrosome-acroplaxome-manchette complex was totally damaged in *Actl7a*-KO mice. The phenotypes of acrosome detachment and defective acroplaxome are consistent with those caused by *Actl7a*/*ACTL7A* with different mutations [10–12, 25]. In addition, the assembly of the manchette is disorganized during spermiogenesis in *Actl7a*-KO mice. The acrosome-acroplaxome-manchette complex defines the specific shape of the sperm head [3]. Disruption of the complex is responsible for the observed aberrant small head sperm in *Actl7a*-KO mice. This further illustrates the crucial role of ACTL7A in spermiogenesis.

Autophagy is an evolutionarily conserved biological process that sustains intracellular homeostasis and survival by degrading waste cytoplasmic contents including defective organelles and proteins [26, 27]. In particular, this process is required for the development and function of the male reproductive system consisting of testosterone biosynthesis, assembly of ectoplasmic specialization, acrosome biogenesis and formation of the cytoskeleton [5, 28]. In this study, a quantitative proteomics analysis of testes from WT and *Actl7a*-KO male mice identifies the PI3K/AKT/mTOR pathway as the most significantly enriched signaling pathway. It is well established that the PI3K/AKT/mTOR signaling pathway plays a vital role in regulating autophagy [17]. Aautophagy is involved in acrosome biogenesis, and knockout of autophagy-related genes in mice results in round-headed spermatozoa with malformed acrosome [5]. Furthermore, autophagy also regulates cytoskeleton organization by degrading some negative regulators of cytoskeleton organization to preserve the proper dynamics of the cytoskeleton. PDLIM1, a member of the PDZ and LIM protein family, is degraded by the autophagy-lysosome pathway in spermatids to maintain a proper level to facilitate cytoskeleton organization, including the microtubules and F-actin networks, the composition of manchette. Therefore, PDLIM1 is a major mediator between autophagy and cytoskeleton organization during spermiogenesis [18]. Moreover, PDLIM1 forms a triple complex with ACTN1 and PALLDA localizing on the manchette [29], and the accumulation of PDLIM1 results in the random orientation of the manchette [18]. Actually, this study confirms that the deletion of ACTL7A results in autophagy inhibition via PI3K/AKT/mTOR signaling pathway activation, and following PDLIM1 accumulation in *Actl7a*-KO mice. This may explain why the development of manchette, the microtubule- and F-actin-based structure, was disrupted. Our findings reveal a new molecular mechanism for small head sperm as a result of the deletion of ACTL7A.

AOA technology can help some couples who fail to fertilize by ICSI owing to oocyte activation deficiency [30, 31]. Previous studies have reported that ICSI-AOA can successfully rescue infertility due to *Actl7a*/*ACTL7A* with different mutations [10–12]. However, there is still some expression of *Actl7a*/*ACTL7A* in these mutants. Here, we report that ICSI-AOA is effective in *Actl7a*-KO mice with the complete absence of ACTL7A protein. This provides a basis for clinical treatment of infertility due to any defective ACTL7A.

## Conclusions

Taken together, the study identifies the new phenotype of small head sperm in the *ACTL7A*-mutated patient. In vitro functional experiments and analysis of the *Actl7a* knockout mouse model reveals that it is caused by damage of the acrosome-acroplaxome-manchette complex. The molecular mechanism is elucidated that autophagy inhibition via PI3K/AKT/mTOR signaling pathway activation leading to PDLIM1 accumulation may be causative to the defective manchette in *Actl7a*-KO mice. ICSI-AOA remains an effective technique to rescue infertility caused by the deletion of ACTL7A. These findings have important implications for clinical treatment and further research into the molecular mechanisms of male infertility.

### Electronic supplementary material

Below is the link to the electronic supplementary material.

Supplementary Material 1

Supplementary Material 2

## Data Availability

Data will be available on request.

## Abbreviations

ACTL7A: Actin-like 7 A
ARPs: Actin-related proteins
ART: Assisted reproductive technologies
ICSI: Intracytoplasmic sperm injection
AOA: Artificial oocyte activation
KO: Knock-out
HTF: Human tubal fluid
PFA: Paraformaldehyde
TEM: Transmission electron microscopy
TMT: Tandem mass tags
LC-MS/MS: Liquid chromatography-tandem mass spectrometry
PMSG: Pregnant mare serum gonadotropin
hCG: Human chorionic gonadotropin
OCC: Oocyte-cumulus complexes
PVDF: Polyvinylidene fluoride
ECL: Enhanced chemiluminescence
DEGs: Differentially expressed genes
IHC: Immunohistochemistry
