# Supplementary figures and images for: Loss of ACTL7A causes small head sperm by defective acrosome-acroplaxome-manchette complex
Source: Reprod Biol Endocrinol. 2023 Sep 4;21:82. doi: 10.1186/s12958-023-01130-5 (PMC10476415; doi:10.1186/s12958-023-01130-5)

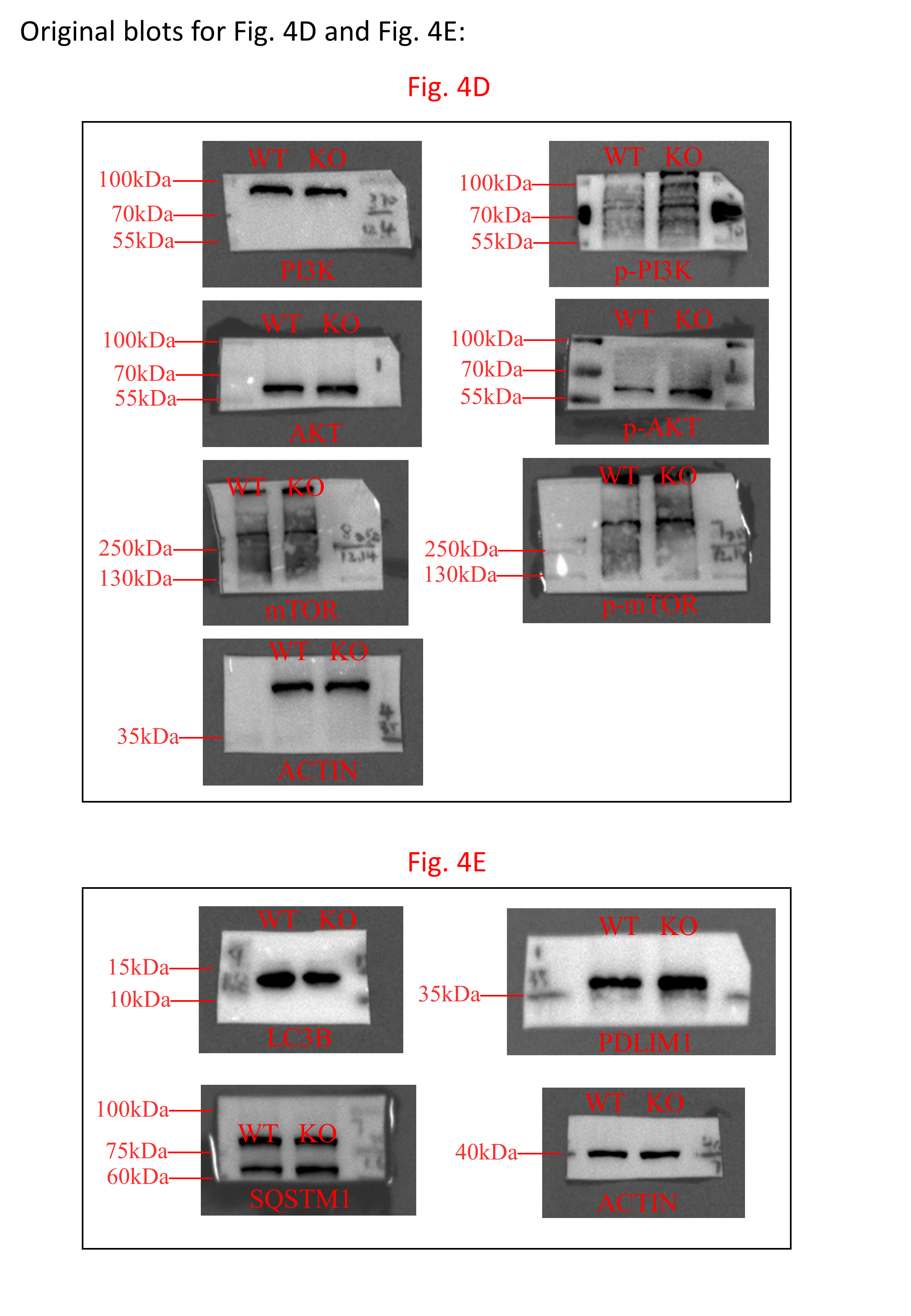

Supplement: Supplementary file 1 — Supplementary Material 1 [file 12958_2023_1130_MOESM1_ESM.png]

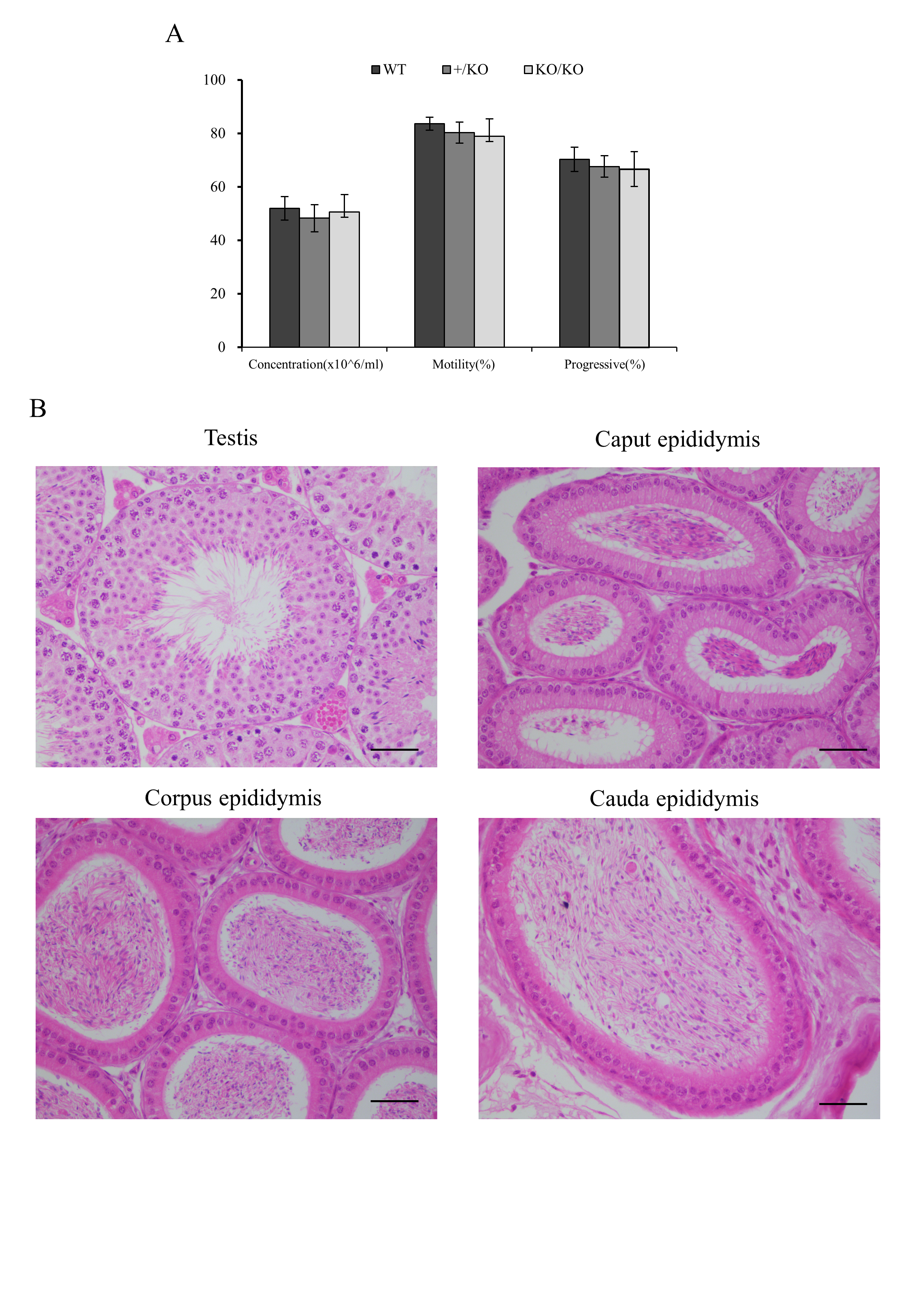

Supplement: Supplementary file 2 — Supplementary Material 2 [file 12958_2023_1130_MOESM2_ESM.png]
